# The use of nano-sized acicular material, sliding friction, and antisense DNA oligonucleotides to silence bacterial genes

**DOI:** 10.1186/s13568-014-0070-7

**Published:** 2014-09-04

**Authors:** Yuya Mitsudome, Mamiko Takahama, Jun Hirose, Naoto Yoshida

**Affiliations:** 1 Department of Biochemistry and Applied Biosciences, University of Miyazaki, 1-1 Gakuen, Kibanadai-Nishi, Miyazaki 889-2192, Japan; 2 Department of Applied Chemistry, University of Miyazaki, 1-1 Gakuen, Kibanadai-Nishi, Miyazaki 889-2192, Japan

**Keywords:** Antisense oligonucleotide DNA, BphD, Gene silencing, β-Galactosidase, β-Lactamase, Sepiolite, Sliding friction, Spo0A

## Abstract

Viable bacterial cells impaled with a single particle of a nano-sized acicular material formed when a mixture containing the cells and the material was exposed to a sliding friction field between polystyrene and agar gel; hereafter, we refer to these impaled cells as penetrons. We have used nano-sized acicular material to establish a novel method for bacterial transformation. Here, we generated penetrons that carried antisense DNA adsorbed on nano-sized acicular material (α-sepiolite) by providing sliding friction onto the surface of agar gel; we then investigated whether penetron formation was applicable to gene silencing techniques. Antisense DNA was artificially synthesized as 15 or 90mer DNA oligonucleotides based on the sequences around the translation start codon of target mRNAs. Mixtures of bacterial cells with antisense DNA adsorbed on α-sepiolite were stimulated by sliding friction on the surface of agar gel for 60 s. Upon formation of *Escherichia coli* penetrons, β-lactamase and β-galactosidase expression was evaluated by counting the numbers of colonies formed on LB agar containing ampicillin and by measuring β-galactosidase activity respectively. The numbers of ampicillin resistant colonies and the β-galactosidase activity derived from penetrons bearing antisense DNA (90mer) was repressed to 15% and 25%, respectively, of that of control penetrons which lacked antisense DNA. Biphenyl metabolite, ring cleavage yellow compound produced by *Pseudomonas pseudoalcaligenes* penetron treated with antisense oligonucleotide DNA targeted to *bphD* increased higher than that lacking antisense DNA. This result indicated that expression of bphD in *P. pseudoalcaligenes* penetrons was repressed by antisense DNA that targeted *bphD* mRNA. Sporulation rates of *Bacillus subtilis* penetrons treated with antisense DNA (15mer) targeted to *spo0A* decreased to 24.4% relative to penetrons lacking antisense DNA. This novel method of gene silencing has substantial promise for elucidation of gene function in bacterial species that have been refractory to experimental introduction of exogenous DNA.

## Introduction

RNA with substantial structure, such as double-stranded RNA, is a poor template for protein synthesis (Lee et al. [1993]; Fire et al. [1998]; Hamilton and Baulcombe [1999]; Elbashir et al. [2001]). Knowledge of this has been used effectively to block translation in cell-free extracts. In vitro, hybrid-arrested translation has been used to prevent the synthesis of specific proteins and has been used to detect recombinant DNA molecules that contain sequences complementary to a given mRNA. Stephenson and Zamecnik ([1978]) first demonstrated that DNA oligonucleotides complementary to the reiterated 3′- and 5′- terminal nucleotides of Rous sarcoma virus 35S RNA inhibit the translation of RNA in a cell-free system; they also prevent virus production by chicken fibroblast grown in tissue cultures (Stephenson and Zamecnik, [1978]; Zamecnik and Stephenson [1978]). This method is a valuable tool for sequence-specific inhibition of gene expression, and also useful for functional genomics, target validation and treatment of diseases (Bennett and Cowsert [1999]; Dean [2001]; Lavery and King [2003]). Presumably, mechanisms involving steric blockage of the ribosomes are responsible for inhibition of transcription (Kurreck [2003]). Izant and Weintraub ([1984]; [1985]) examined the effects of complementary antisense RNA on the expression of the herpes simplex virus I thymidine kinase (TK) gene in mouse cells. Microinjection of recombinant DNA plasmid that directs transcription of the TK gene with reverse polarity causes the mouse cells themselves to produce the antisense RNA. TK activity is diminished 4- to 5-fold in cells that produce such antisense RNA; this finding indicated that antisense nucleotides may be used to inhibit expression of specific genes. Molecular biologists use antisense DNA or RNA fragments as powerful tools to block expression of specific target genes, and thereby assess gene function.

Upon bacterial gene silencing method, Jayaraman et al. ([1981]) synthesized a series of deoxyribooligonucleoside methylphosphonates, which are nonionic oligonucleotide analogs. The base sequences of these compounds are complementary to the Shine-Dalgarno sequence found at the 3′ end of bacterial 16S rRNA. These oligonucleoside methylphosphonates inhibit both protein synthesis and colony formation by mutant *E. coli*; importantly, these permeable mutants contain negligible quantities of lipopolysaccharide. Experiments were done on the growth of *E. coli* in broth in the presence of the oligonucleotide methylphosphonate; culture growth was inhibited up to 50%, relative to controls, by the oligonucleotide. Nakashima *et al*. ([2006]) used *E. coli* to produce a gene silencing effect on bacterial species; they found that 100 bp of paired-termini antisense RNA enable gene silencing to be dramatically effective than short length antisense RNA.

For an oligonucleotide to display an antisense effect, it must be stable until it forms a double helix with the target mRNA. Phosphodiester oligo DNA (D-oligo DNAs), which are frequently used as antisense oligonucleotides, may be digested by nuclease. To stabilize antisense oligonucleotides, different types of oligonucleotide analogs (*e.g.,* phosphorothioates oligonucleotide DNA, methylphosphoate oligonucleotide DNA) have been developed (Akhtar et al. [1991]; Zhao et al. [1993]; Verma and Eckstein [1998]). However, analogs of D-oligo DNA are disadvantageous for gene silencing because they are very costly to synthesize. Additionally, other data indicate that antisense DNA exhibits efficacy in vivo when administered at the relatively high concentration of 5 mM (Whitesell et al. [1991]). The high doses are necessary in vivo because of the susceptibility of normal DNA oligomers to rapid degradation by serum nucleases. More likely candidates for in vivo applications are modified oligonucleotides that are resistant to nucleases because the nucleotide structure itself is altered (e.g., alkylphosphonates, phosphorothioates, 2′-*O*-alkylnucleotides) or because protecting groups are present at the 3′ and 5′ ends of DNA oligomers (Wickstrom [1992]). Which such modifications most effectively balance cost, in vivo and intracellular mobility, stability, and most importantly, binding affinity and specificity, remain to be determined.

An antisense oligonucleotide is preferably between 15 and 30 mer in length and specifically hybridizes with an mRNA around the translation start codon (Uhlmann and Peyman [1990]; Good et al. [2001]). Recently, Nakashima *et al*. successfully repressed the β-galactosidase gene by engineering expression of a 100 mer antisense oligonucleotide that was complementary to the mRNA near the ribosome binding site and the translation start codon (Nakashima and Tamura, [2009]).

**Figure 1:**
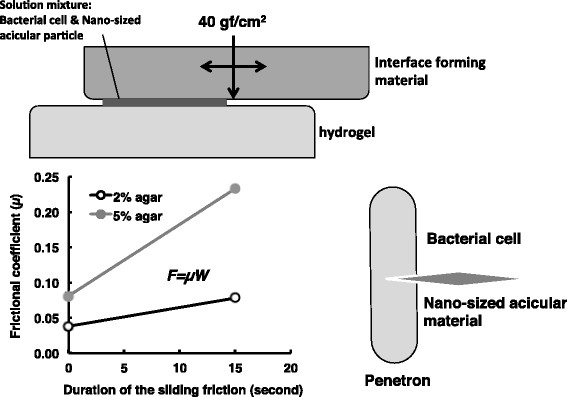
**Schematic representation of the Yoshida effect.** The Yoshida effect is defined as the formation of complexes called penetrons, which are bacterial cells, each impaled by a single nano-sized acicular material in a friction field formed at a hydrogel interface. The hydrogel, interface forming material, nano-sized acicular material, bacterial cells, sliding friction, and an energy source to provide the friction force are each essential to the formation of penetrons. The hydrogel (e. g., agar, gellan gum, κ-karagenan) involved shear stress at more than 2.1 N. The interface forming material could comprise polymer material such as polystyrene, polyethylene, or acrylonitrile butanediene rubber. The optical vertical reaction force against hydrogel was around 40 gf/cm^2^. Multi-walled carbon nanotube, maghemite, or α-sepiolite are each nano-sized acicular materials that can generate a Yoshida effect. The sliding friction force can be represented with the following formula: F = μW, where μ and W denote the frictional coefficient and the vertical reaction force, respectively. When using 2 and 5% agar hydrogel, frictional coefficient value increased 0.038 to 0.078 and 0.081 to 0.233 respectively by given sliding stimulus for 15 seconds. The rapid increase in frictional resistance was essential for the Yoshida effect.

As shown in Figure 1, a bacterial cell impaled by a nano-sized acicular material (penetron) was formed when bacterial cells and nano-sized acicular materials were exposed to a friction field in hydrogel. This phenomenon is called the Yoshida effect (Yoshida and Sato [2009]). Penetrons readily take up exogenous DNA; consequently, penetrons are applicable to a genetic transformation technique called tribos transformation (Yoshida and Sato [2009]). Transformation efficiency of *E. coli* penetrons is 10^7^ colony forming units (cfu) per 1 μg of plasmid DNA (Yoshida et al. [2007]). Nano-sized acicular materials, e.g., chrysotile and sepiolite, have high affinity for nucleic acid. Notably, DNA adsorbed on nano-sized acicular materials is protected from enzyme-mediated nucleolytic degradation and remains stable (Somiya et al. [2012]).

We reasoned that penetrons that had been mixed with artificially synthesized antisense DNA could be used for bacterial gene silencing techniques. In this study, to investigate whether bacterial penetron was utilized to gene silencing technique, we introduced artificially synthesized antisense oligonucleotide DNA into bacterial cells by using sliding friction and sepiolite. We used *Escherichia coli* JM109 harboring pUC18 DNA and *Pseudomonas pseudoalcaligenes* KF707 to represent Gram-negative bacteria. We synthesized 15 and 90 mer antisense DNA oligonucleotides; each was targeted to the translation start codon of β−galactosidase α-fragment gene or β−lactamase gene coded in pUC18 DNA. Silencing of the β−galactosidase α-fragment was evaluated by measurement of enzyme activity; silencing of β−lactamase was evaluated by numerical comparisons of ampicillin resistant colonies. *Pseudomonas pseudoalcaligenes* KF707 was isolated by Furukawa and Miyazaki ([1986]) as a biphenyl degrading bacterium (1986). The genes associated with biphenyl metabolism and their functions are well studied (Furukawa and Miyazaki [1986]; Furukawa and Arimura, [1987]; Taira et al. [1992]). We synthesized 15 and 90 mer antisense DNA oligonucleotides; each was targeted to the 2-hydroxy-6-oxo-6-phenylhexa-2,4-dienoate hydrolase gene (*bphD*). We then evaluated expression of *bphD. Bacillus subtilis* str. 169 (Kunst et al. [1997]) represents Gram-positive spore-forming bacterium. We also synthesized antisense DNA oligonucleotides (15 and 90 mer) targeting the translation start codon of sporulation initiation protein gene (*Spo0A*). Gene silencing of *spo0A* was evaluated by spore formation rate and accumulation of dipicolinic acid in spores.

## Materials and methods

### Bacterial strains and growth condition

*Escherichia coli* JM109 (el4^−^, *rec*A1, *end*A1, *gyr*A96, *thi*-1, *hsd*R17, *sup*E44, *rel*A1, ∆(*lac-pro*AB), [F’ , *tra*D36 *pro*AB, *lac*I^q^ Z∆M15]) harboring pUC18 DNA was grown aerobically in Luria-Bertani (LB) broth containing 50 μg/ml ampicillin at 30°C for 18 hrs (Sambrook et al. [1989]; Shiloach et al. [1996]).

*Pseudomonas pseudoalcaligenes* KF707 which has been deposited in the NITE Patent Microorganisms Depositary under the accession number FERM P-8297 was grown in a defined medium (pH 7.0) containing (in grams per liter): K_2_HPO_4_, 4.3; KH_2_PO_4_, 3.4; (NH_4_)_2_SO_4_, 2.0; MgCl_2_, 0.16; MnCl_2_・4H_2_O, 0.001; FeSO_4_・7H_2_O, 0.006; CaCl_2_・2H_2_O, 0.026; and Na_2_MoO_4_・2H_2_O, 0.002. For 1.5% agar plating medium (Wako Chemical Co., Japan), biphenyl was introduced as a vapor by placing crystals on the lid of each petri dish. Each dish was sealed with polyethylene tape. Strain KF707 cells were inoculated onto agar plating medium and incubated at 30°C for 4 days (Furukawa and Miyazaki [1986]).

*Bacillus subtilis* str.168 (Kunst et al. [1997]) was cultured on LB agar medium that contained 1% glucose at 37°C for 18 hrs.

### Preparation of sepiolite solution

Sepiolite was used as the nano-sized acicular material in each experiment to form penetrons. Sepiolite particles (Wako Chemical Co., Japan) suspended in distilled water at final concentrations of 50 μg/ml or 1 mg/ml for Gram-negative and Gram-positive bacteria respectively were incubated in suspension at 60°C for 24 hrs, and the sepiolite solution was then used.

### Design of antisense DNA oligonucleotides

To evaluate the effects on gene silencing of DNA oligonucleotides in *E. coli*, two mRNAs—β-lactamase (*bla*) and β-galactocidase (*lacZα*)—were targeted. To assess the effects in *Pseudomonas* and *Bacillus*, the mRNAs encoding hydrolase (*bphD*), which is associated with biphenyl metabolism (Triscari-Barberi et al. [2012]) and sporulation initiation protein (*spo0A*) were targeted, respectively. The sequence of each 15 and 90 bp DNA oligonucleotide was designed based on the sequence around the translation start codon in the respective targeted mRNA. The sequence of each antisense DNA oligonucleotide DNA and the respective abbreviations are listed in Table 1.

**Table 1:** Sequence of each DNA oligonucleotide used in this study

**Strain**	**Target gene**	**Name of antisense oligonucleotide**	**Accession no.**	**Position**	**Sequence of antisense oligonucleotide DNA (5′-3′)**	**Length of antisense oligonucleotide DNA (bp)**
*Escherichia coli* JM109 (pUC18)	β-lactamase (*bla*)	B-LAC15	L08752	876-891	ACT***CAT***ACTCTTCCT	15
		B-LAC90		851-940	AGGCAAAATGCCGCAAAAAAGGGAATAAGGGCGACACGGAAATGTTGAATACT***CAT***ACTCTTCCTTTTTCAATATTATTGAAGCATTTAT	90
	β-galactosidase (*lacZα*)	LacZ15		221-207	GGT***CAT***AGCTGTTTC	15
		LacZ90		185-274	CTGCAGGTCGACTCTAGAGGATCCCCGGGTACCGAGCTCGAATTCGTAATCATGGT***CAT***AGCTGTTTCCTGTGTGAAATTGTTATCCGCT	90
*Pseudomonas pseudoalcaligenes* KF707	Hydrolase (*bphD*)	BphD15	X66123	178-164	GTGCGGT***CAT***TTTTC	15
		BphD90		231-142	GGTAGAACTTTCGGTGAGTGCGGT***CAT***TTTTCATCCTTTAAGTGAGTGGAGTGGAAACTGGTCAGGCGCAGCGCTTCATGCCGCGCCGGG	90
*Bacillus subtilis* str.168	Sporulation initiation protein (*spo0A*)	Spo0A15	AL009129	2518821-2518835	CTC***CAC***GTTTCTTCC	15
		Spo0A90		2518768-2518857	CTTAACAGGCTTACCAGCTCTCGATTATCATCAGCAACACAAACTTTAATTTTCTC***CAC***GTTTCTTCCTCCCCAAATGTAGTTAACAGGA	90

Bold italic letter indicates sequence complementary to the translation codon (AUG or GUG) in a target mRNA.

### Introduction of antisense oligonucleotide DNA into *E. coli* penetrons and evaluation of gene silencing effect on β-lactamase

A 50 μl aliquot of sepiolite solution containing 0.2 μM of antisense oligonucleotide DNA (B-LAC15, B-LAC90), 200 mM NaCl, and an equivalent volume of *E. coli* culture broth were streaked onto the surface of 2% agar hydrogel containing LB contents and 100 μg/ml of ampicillin. Sliding friction between a polystyrene streak bar (SARSTED, Germany) and agar hydrogel was applied to these solution mixtures for 60 sec. The agar hydrogel was incubated at 37°C for 18 hrs, and then ampicillin resistant colonies were counted. The number of ampicillin resistant colony formed on the agar hydrogel treated by same method without antisense oligonucleotide DNA was used as the 100% control.

### Introduction of antisense DNA oligonucleotides into *E. coli* penetrons and evaluation of gene silencing effect on β-galactosidase α-fragment

A 100 μl mixture containing 50 μl of sepiolite solution with 0.2 μM of antisense DNA oligonucleotide (LacZ15, LacZ90), 200 mM NaCl and 50 μl of *E. coli* culture broth was streaked onto the surface of a 2% agar hydrogel containing 1 mM β-D-thiogalactopyranoside (IPTG) and 200 mM NaCl. Each of these mixtures and control mixtures that were identical except that they lacked any antisense oligonucleotides were individually exposed to the sliding friction between a polystyrene streak bar (SARSTED) and agar hydrogel for 60 sec. Each agar hydrogel coated with cells was then incubated at 37°C for 3 hrs; *E. coli* penetrons on agar hydrogel were then resuspended in 2 ml of NaCl solution. A standard colorimetric method (Miller, [1972]; Griffith and Wolf [2002]) was then used to measure β-galactosidase activity in control and oligonucleotide-treated *E. coli* penetrons. Values of β-galactosidase activity in the control *E. coli* penetrons were designated as the 100% standard.

### SDS-PAGE and analysis of protein bands

A 100 μl mixture containing 50 μl of sepiolite solution with 0.2 μM of antisense DNA oligonucleotide (LacZ15, LacZ90), 200 mM NaCl and 50 μl of *E. coli* culture broth was streaked onto the surface of a 2% agar hydrogel containing 1 mM IPTG and 200 mM NaCl. Again, each mixture was exposed to the sliding friction field between a streak bar and agar hydrogel for 60 sec. The cell-coated agar hydrogels were each incubated at 37°C for 3 hrs; control and oligonucleotide-treated *E. coli* penetrons were collected from the surface of the agar hydrogels with 2 ml of 200 mM NaCl and a cell scraper. Proteins were extracted from collected *E. coli* penetrons by sonication and analyzed by SDS-PAGE according to the Leammli protocol (Laemmli, [1970]). Each proteins gel was stained and then destained; electrophoresis image analysis software (NIH ImageJ, Version 1.41) (http://rsb.info.nih.gov/ij/) was then used to measure the concentration of protein in each band that corresponded to β-galactosidase.

### Introduction of antisense oligonucleotide DNA into *P. pseudoalcaligenes* penetrons and evaluation of gene silencing effect on *bphD* expression

Fresh *P. pseudoalcaligenes* cells (10 mg/ml) were suspended in 50 μl of sepiolite solution containing 200 mM NaCl and 0.2 μM of antisense DNA oligonucleotide (bphD15 or bphD90) or no oligonucleotides. Each 50 μl mixture was spread onto the surface of a 2% agar hydrogel containing 200 mM NaCl. Each mixture was exposed to sliding friction between a streak bar and agar hydrogel for 60 sec to form *P. pseudoalcaligenes* penetrons. Control and oligonucleotide-treated penetrons were collected from the surface of each agar hydrogel with 2 ml of 200 mM NaCl and a cell scraper. Collected *P. pseudoalcaligenes* penetrons were washed with 200 mM phosphate buffer (pH 7.0); that was used to adjust the turbidity of each sample of *P. pseudoalcaligenes* penetrons to an absorbance of 0.2 at 600 nm. Each 1.0 ml suspension of *P. pseudoalcaligenes* penetrons was added to a respective 20 ml volume of 0.1 μM biphenyl solution; each such mixture was then incubated at 30°C. After a 40 min and 80 min incubation, the absorbance value at 403 nm of each centrifuged reaction mixture was determined with a spectrophotometer (UV-1800, Shimazu, Japan) (Triscari-Barberi et al. [2012]).

### Introduction of antisense oligonucleotide DNA into *B. subtilis* penetrons and evaluation of gene silencing effect on *spo0A* expression

Fresh *B. subtilis* vegetative cells (10 mg/ml) were suspended in 50 μl of sepiolite solution containing 200 mM NaCl and 0.2 μM of antisense Spo0A15 or Spo0A90 DNA oligonucleotide or no oligonucleotides. The 50 μl mixture was spread onto the surface of a 5% agar hydrogel containing 10 g/L NaCl, 0.016 g/L MnCl_2_, 0.12 g/L MgSO_4_, 0.016 mg/L FeSO_4_, 0.11 mg/L CaCl_2_, and 8.3 μl/L HCl. The mixture was exposed to the sliding friction between the streak bar and agar hydrogel for 60 sec to form penetrons. The *B. subtilis* penetrons formed on the surface of the agar hydrogel were incubated at 30°C for 72 hrs, and were then collected from the surface of the agar hydrogel with 2 ml of 200 mM NaCl and a cell scraper. The number of vegetative cells and spores was counted under a confocal microscope (ZEISS, Germany) and spore formation rate (spores/spores + vegetative cells) was calculated.

To alternatively evaluate the effect of antisense oligonucleotide DNA on spore formation rate, we measured the amount of dipicolinic acid (DPA) production by *B. subtilis* penetrons (Janssen et al. [1958]; Rotman and Fields [1968]). The *B. subtilis* penetrons formed on the surface of the agar hydrogel were incubated at 30°C for 72 hrs, and were collected from the surface of the agar hydrogel with 2 ml of 200 mM NaCl and a cell scraper. The *B. subtilis* penetrons were washed by centrifugation, suspended in 1 ml of distilled water, boiled for 20 min, cooled in ice for 15 min and then exposed to DPA extract solution. A spectrophotometer (UV-1800, Shimazu, Japan) was then used to measure the absorbance at 440 nm of a mixture of 0.4 ml of centrifugated DPA extract solution, 0.2 ml of distilled water, and 0.2 ml of coloring reaction solution containing 1.0 g/L L-Cysteine, 6.8 g/L FeSO_4_・7H_2_O, 3.2 g/L (NH_4_)_2_SO_4_, and 50 mM sodium acetate buffer (pH4.6). Reagent grade DPA (Wako Chemical Co., Japan) was used as the standard.

## Results

### Evaluation of gene silencing effect on β-lactamase

**Figure 2:**
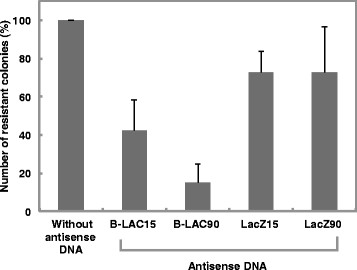
**Gene silencing effects of antisense DNA oligonucleotides (B-LAC15, 90) on β-lactamase expression in*Escherichia coli*JM109 (pUC18).** Values indicate the means ± standard deviation of three independent experiments.

The number of ampicillin resistant colonies derived from penetrons exposed to the antisense DNA oligonucleotides, B-LAC15 or B-LAC90, decreased to 42% or 15% of the controls respectively (Figure 2). These results indicated that the antisense oligonucleotides bound to the complementary region of the mRNA encoding β-lactamase, which catalyzes the hydrolysis of the β-lactam ring in the ampicillin molecule, and that the oligonucleotides inhibited translation of the bound mRNAs. The oligonucleotides LacZ15 and LacZ90, which were synthesized as antisense oligonucleotides targeted to the mRNA encoding the β-galactosidase α-fragment, were not associated with the mRNA encoding β-lactamase; nevertheless the number of ampicillin resistant colonies derived from penetrons exposed to LacZ15 or LacZ90 decreased to 72% of the controls. The mRNA encoding β-lactamase assumes to involve semi-complementary sequence with oligonucleotide LacZ15 and LacZ90.

### Evaluation of gene silencing effect on β-galactosidase α-fragment

**Figure 3:**
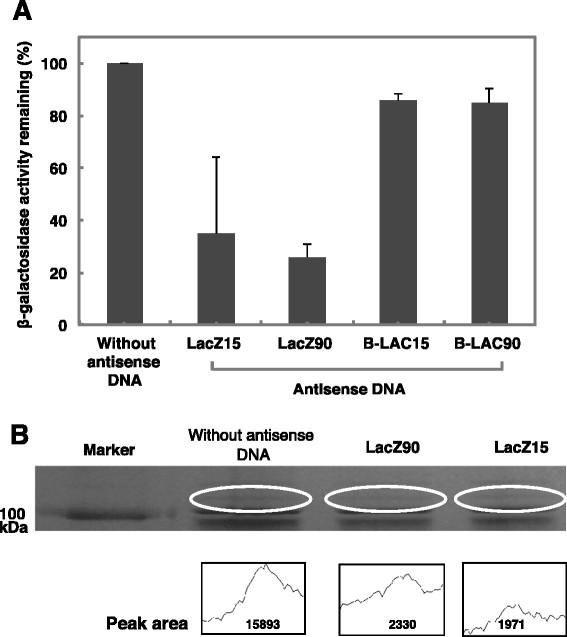
**Gene silencing effects of antisense DNA oligonucleotides (LacZ15, 90) on β -galactosidase activity in*Escherichia coli*JM109 (pUC18).(A)** Values indicate the means ± standard deviation of three independent experiments. **(B)** Comparison of β -galactosidase band intensity on SDS-PAGE gels from *E. coli* bearing or lacking antisense DNA (LacZ15, 90). The peak area indicates the intensity of Coomassie-stained protein bands that correspond to β-galactosidase.

β-galactosidase activity in *E. coli* penetrons that had been exposed to antisense DNA oligonucleotides (LacZ15 or LacZ90) was reduced to 38.4 and 26.0% of that in control *E. coli* penetrons respectively (Figure 3A). These antisense DNAs would have inhibited translation of targeted mRNA by binding complementary sequences in the 5’-region of the targeted mRNA and resulted in depression of β-galactosidase activity. The β-galactosidase activity in *E. coli* penetrons that were exposed to B-LAC15 or B-LAC90 was reduced to 84% of that in control *E. coli* penetrons. This result was attributed to the possibility that the mRNA encoding β-galactosidase carried complementary sequences similar to the B-LAC15 and B-LAC90 sequences.

Coomassie-stained protein bands that corresponded to the β-galactosidase expressed in *E. coli* penetrons into which LacZ90 or LacZ15 had been injected exhibited 2330 and 1971 as peak intensities respectively (Figure 3B). In the mean time, the protein bands corresponding to the β-galactosidase expressed in control *E. coli* penetrons exhibited a higher intensity at 15893. This result indicated that β-galactosidase expression in *E. coli* penetrons in which LacZ90 or LacZ15 had been injected was depressed to 14.7 and 12.4% of that in control *E. coli* penetrons respectively.

### Evaluation of gene silencing effect on hydrolase

**Figure 4:**
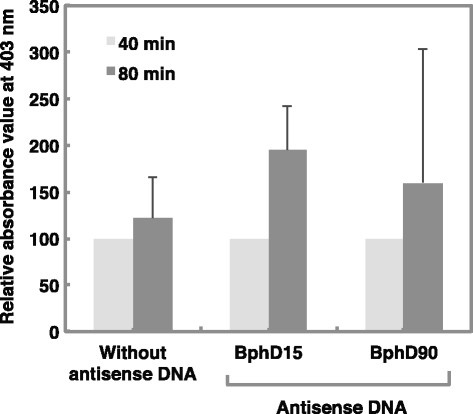
**Gene silencing effects of BphD15 and 90 antisense oligonucleotide DNA on hydrolase activity in*Pseudomonas pseudoalcaligenes*KF707.** Values indicate the means ± standard deviation of three independent experiments.

The biphenyl solution added to *P. pseudoalcaligenes* penetrons in which antisense DNA oligonucleotide DNA had been injected and that added to control *P. pseudoalcaligenes* penetrons were incubated at 30°C (reaction solution). Absorbance value at 403 nm of reaction solution after a 40 min incubation was defined as 100%. Absorbance value at 403 nm of the control reaction solution after an 80 min incubation was 122%. Absorbance values at 403 nm of the BphD15 and BphD90 reaction solutions after an 80 min incubation were 195 and 160% respectively (Figure 4). The results showed that the gene silencing cause by the 15 mer oligonucleotide was stronger than that cause by the 90 mer oligonucleotide.

### Evaluation of gene silencing effects on sporulation formation rates

**Figure 5:**
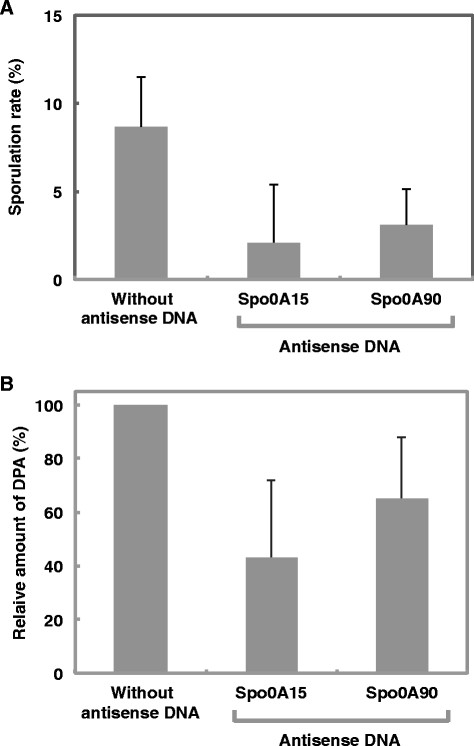
**Gene silencing effects of Spo0A15 and 90 antisense oligonucleotides DNA on (A) spore formation and (B) dipicolinic acid accumulation in*Bacillus subtilis*str. 168.** Values indicate the means ± standard deviation of three independent experiments.

Spo0A is a signal protein involved in initiation of *Bacillus* sp. sporulation. Spo0A is activated by phosphorylation and serves as a translation regulation factor for one set of sporulation mRNAs (Errington, [1993]). Phosphorylation-mediated activation of spo0A is essential for switching of vegetative cells to spore cells, thus gene silencing of *spo0A* should cause repression of differentiation to spore cells. Spore formation rates of *B. subtilis* penetrons injected with antisense oligonucleotides (Spo0A15 or Spo0A90) was repressed to 24.4% and 35.8% of that of control *B. subtilis* penetrons respectively (Figure 5A). Alternatively, the amount of dipicolinic acid (Janssen et al. [1958]; Rotman and Fields [1968]) production of *B. subtilis* penetrons in which antisense oligonucleotide Spo0A15 and Spo0A90 was injected was repressed to 42.2 and 65.3% of control *B. subtilis* penetrons (Figure 5B). Presumably, these antisense DNAs bound to complementary sequences within the targeted *spo0A* mRNA and inhibited the translation of spo0A, thereby resulting in depression of spore formation in *B. subtilis* penetrons.

## Discussion

The most common methods used to explore gene function involve gene deletion (gene knockout) or inactivation of target mRNA (gene knockdown). Antisense methods are among the most commonly used for such research purposes. Antisense molecules, as experimental materials, involve ribozymes and antisense RNA generated from expression vectors. Here, we focused on a method involving artificially synthesized DNA oligonucleotides. Antisense RNAs expressed from vectors have lead to many achievements in biochemical research. However, construction of such expression vectors is technically time consuming and low amount of antisense RNA can be expressed is still problematic. The advantages of our proposed gene silencing technique, which involves antisense DNA oligonucleotides, are that specially prepared equipment and special techniques are not required and that many genes can be targeted. In proposed gene silencing method using nano-sized acicular material and antisense DNA, penetrons that lack antisense DNA or penetrons with antisense DNA complementary to off-target genes must be used as controls for any experiments.

β−lactamase and β−galactosidase α-fragment genes are encoded in pUC18 DNA, and expression of the genes was induced by ampicillin, which bears a β−lactam ring, and IPTG, respectively. Comparisons among effects on gene silencing of β−lactamase and β−galactosidase α-fragment and resulting from oligonucleotides of different lengths demonstrated that the 90 mers were more effective than 15 mers. This result may depend on secondary structure of targeted mRNA or stability of injected antisense oligonucleotide DNA.

The *P. pseudoalcaligenes* strain used in our experiments was isolated from soil near a biphenyl-producing factory in Kitakyushu, Japan (Furukawa and Miyazaki [1986]). *P. pseudoalcaligenes* KF707 can degrade 4, 4′-dichlorobiphenyl quickly, but it degrades 2, 5, 2′, 5′-tetrachlorobiphenyl poorly (Mondello et al. [1997]). A catabolic pathway responsible for biphenyl degradation by *P. pseudoalcaligenes* KF707 has been proposed by Furukawa and Miyazaki ([1986]). This strain expresses 2, 3-dioxygenase, which introduces molecular oxygen at the 2, 3-position of a nonchlorinated or less-chlorinated ring in polychlorinated biphenyl to produce a dihydrodiol (Kimura et al. [1997]). The dihydrodiol is then dehydrogenated to a 2, 3-dihydroxybiphenyl by a dihydrodiol dehydrogenase. The 2, 3-dihydroxybiphenyl is then cleaved at the 1,2-position by a dihydroxybiphenyl dioxygenase to produce 2-hydroxy-6-oxo-6-phenylhexa-2,4-dienoate (HPDA). The *meta* cleavage compound, HPDA, is then hydrolyzed to the chlorobenzoic acid and 2-oxopent-4-enoate by a hydrolase which is the product of *bphD*. Here we measured the activity of this hydrolase by monitoring the conversion of the HPDA to benzoic acid by measuring the decrease in absorbance at 403 nm. When biphenyl was degraded by *P. pseudoalcaligenes* penetrons bearing the *bphD* antisense DNA target, the HPDA-associated absorbance values at 403 nm were significantly higher than those of the control *P. pseudoalcaligenes* penetrons. These data indicated that antisense DNA inhibited translation of *bphD* mRNA and depressed expression of hydrolase (bphD), and that this inhibition resulted in the accumulation of HPDA.

Currently, the nine genes involving *spo0A* are known as the genes associated with initiation of sporulation in *B. subtilis* (Tan and Ramamurthi [2014]). If any one of the sporulation proteins is nonfunctional because of genetic mutation, sporulation does not initiate even under starvation conditions. Activation of the transcription factor encoded by spo0A is essential for initiation of sporulation in *B. subtilis* (LeDeaux et al. [1995]). External signals that result from environmental stress induce phosphorylation of Spo0A, and phosphorylated Spo0A activates the σ^F^, σ^E^ subunit of RNA polymerase (Molle et al. [2003]). As expected, the sporulation rate among *B. subtilis* penetrons bearing Spo0A15 or 90 oligonucleotides was lower than that among controls. We presumed that the Spo0A15 and 90 antisense DNA inhibited translation of *spo0A* mRNA and repressed initiation of sporulation. Dipicolinic acid (DPA) was accumulated intracellularly during sporulation, and we could measure DPA accumulation to evaluate sporulation rates. Notably, spo0A15 or spo0A 90 antisense DNA decreased DPA accumulation and repressed endospore formation. The effects of the 15 mer on expression of hydrolase and spo0A were greater than those of the 90 mer. These results were consistent with results reported by many other researchers (Krötz et al. [2003]; Falzarano et al. [2014]).

It is noteworthy that the effects on gene silencing caused by the antisense DNA and nano-sized acicular materials were transient, thus this method affected only resting cells. In these experiments, we used antisense DNA oligonucleotides that were chemically stable and reasonable to synthesize. Many published results demonstrate that the length of antisense oligonucleotides that are effective for gene silencing ranges from 15 to 25 residues (Uhlmann and Peyman [1990]; Mizuta et al. [1999]). The antisense DNAs with sequences complementary to sequences around the target AUG translation initiation codon should provide favorable results for gene silencing. A mechanism by which antisense DNA oligonucleotides are released from α-sepiolite was proposed previously (Yoshida and Ide [2008]). Our novel gene silencing method described in this paper was more effective with Gram-negative bacteria than Gram-positive bacteria. We suspect that Gram-positive penetrons were harder to generate than Gram-negative penetrons because the cell wall of Gram-positive bacteria is thicker than that of Gram-negative bacteria. Until now, researchers have used *E. coli* mutants with increased permeability properties to introduce antisense DNA oligonucleotides when evaluating the gene silencing effects of antisense DNA (Jayaraman et al. [1981]). The advantage of penetron technology for gene silencing is that the technique is simple and applicable to different bacterial species, and many such species have been unable to directly uptake oligonucleotide DNA until now. Once the experimental protocol is established for many bacterial species, the proposed gene silencing technique will be a powerful tool for the elucidation of physiological functions of biological molecules. In any case, our findings demonstrated the substantial potential of antisense DNA as a new class of gene silencing molecules for not only bacteriology but also molecular biology, physiology, developmental biology involving clinical research.

## Competing interest

The authors declare that they have no competing interest.

## Author’s contribution

YM carried out the measurement of β-lactamase and β-galactosidase activity in *E. coli* and sporulation rate in *B. subtilis*. MT carried out the measurement of hydrolase activity in *P. pseudoalcaligenes.* JH participated in the design of the study on gene silencing in *P. pseudoalcaligenes*. NY participated in the design, coordination of the study and contributed in the drafting of the manuscript. All authors read and approved the final manuscript.
